# Supplementary material for: FOXP2 exhibits projection neuron class specific expression, but is not required for multiple aspects of cortical histogenesis
Source: eLife. 2019 May 17;8:e42012. doi: 10.7554/eLife.42012 (PMC6561705; doi:10.7554/eLife.42012)
Supplement: Transparent reporting form [file elife-42012-transrepform.docx]

***eLife’s* transparent reporting form**

We encourage authors to provide detailed information *within their submission* to facilitate the interpretation and replication of experiments. Authors can upload supporting documentation to indicate the use of appropriate reporting guidelines for health-related research (see [EQUATOR Network](http://www.equator-network.org/%20)), life science research (see the [BioSharing Information Resource](https://biosharing.org/)), or the [ARRIVE guidelines](http://www.plosbiology.org/article/info:doi/10.1371/journal.pbio.1000412) for reporting work involving animal research. Where applicable, authors should refer to any relevant reporting standards documents in this form.

If you have any questions, please consult our Journal Policies and/or contact us: [editorial@elifesciences.org](mailto:editorial@elifesciences.org).

**Sample-size estimation**

- You should state whether an appropriate sample size was computed when the study was being designed
- You should state the statistical method of sample size computation and any required assumptions
- If no explicit power analysis was used, you should describe how you decided what sample (replicate) size (number) to use

Please outline where this information can be found within the submission (e.g., sections or figure legends), or explain why this information doesn’t apply to your submission:

Power analyses were not performed prior to the experiments in this study. The sample sizes were decided based on published studies that reported changes in cortical phenotypes of a similar nature to those measured in the present study. Within the methods section, we have included an explicit statement describing this as well as references to published studies that contain similarly small sample sizes.

**Replicates**

- You should report how often each experiment was performed
- You should include a definition of biological versus technical replication
- The data obtained should be provided and sufficient information should be provided to indicate the number of independent biological and/or technical replicates
- If you encountered any outliers, you should describe how these were handled
- Criteria for exclusion/inclusion of data should be clearly stated
- High-throughput sequence data should be uploaded before submission, with a private link for reviewers provided (these are available from both GEO and ArrayExpress)

Please outline where this information can be found within the submission (e.g., sections or figure legends), or explain why this information doesn’t apply to your submission:

-We defined biological replicates (N) as the number of separate animals used in each experiment. Each data point in our bar graphs represents a single animal with one to three cortical tissue sections quantified and averaged per animal per experiment, and an identical number of sections analyzed for each animal within an experiment. For each experiment, the number of animals (N) is reported in its associated figure legend. Littermate pairs from a minimum of two separate litters contributed to each genotype group in each experiment.

-Technical replicates – Immunohistolological staining procedures in this study were repeated on separate days, at least twice, and no quantitative or qualitative differences were observed between procedures repeated on separate days.

-No outliers were detected or excluded from the experiments in this study. All animals that entered the experiments reported were included in the final manuscript.

**Statistical reporting**

- Statistical analysis methods should be described and justified
- Raw data should be presented in figures whenever informative to do so (typically when N per group is less than 10)
- For each experiment, you should identify the statistical tests used, exact values of N, definitions of center, methods of multiple test correction, and dispersion and precision measures (e.g., mean, median, SD, SEM, confidence intervals; and, for the major substantive results, a measure of effect size (e.g., Pearson's r, Cohen's d)
- Report exact p-values wherever possible alongside the summary statistics and 95% confidence intervals. These should be reported for all key questions and not only when the p-value is less than 0.05.

Please outline where this information can be found within the submission (e.g., sections or figure legends), or explain why this information doesn’t apply to your submission:

-Parametric statistical tests (ANOVA and unpaired t-tests) are reported for most experiments, given their power to detect differences (which we did not observe) in comparisons involving small sample sizes. In other words, Parametric tests were chosen, given the inability of non-parametric tests (Mann-Whitney test) to yield statistical significance when group sizes are less than N=4. In this context, we ran non-parametric tests where the tests were adequately powered to detect differences (Figure 3C, D).

-We acknowledge that the small sample sizes in our experiments comes with the caveat that the normality assumption inherent to parametric statistical tests could not be tested, but, to our knowledge, these tests were the only tests that could yield significant results with our sample sizes, yet failed to do so.

-Raw data (quantification of cell counts for each animal) are presented in the bar graphs for all experiments, and corresponding N is stated in the figure legend. All bar graphs are presented as mean ± standard error of the mean. The specific mean and standard error values are explicitly stated in the text of the results section.

**Table 1. Table of Experiments, Statistical Tests, Group Sizes, and p-values**

| Figures | Experiment | Groups Compared | Statistical Test | Group Sizes | P-Value |
| --- | --- | --- | --- | --- | --- |
| 2C, 2E, 2G | Co-expression of MetGFP, CCK, or Fog2 by Ntsr1-cre^+^ corticothalamic neurons at P14 | 1. WT  (Ntsr1-Cre; Foxp2^+/+^ RosaTdTom^Fx/+^ Met^GFP)^  2. cHET  (Ntsr1-Cre; Foxp2^Fx/+^ RosaTdTom^Fx/+^ Met^GFP)^  3. cKO  (Ntsr1-Cre; Foxp2^Fx/Fx^ RosaTdTom^Fx/+^ Met^GFP)^ | One-way ANOVA | 1. WT; N=3  2. cHET; N=2  3. cKO; N=3 | MetGFP (Fig. 2C)  p=0.4419  CCK (Fig. 2E)  p=0.5016  FOG2 (Fig. 2G)  p=0.3163 |
| 3C, 3D | Quantification of FOG2+ (CT) or CTIP2+/FOG2- (PT) neurons at P0 | 1. Foxp2Fx  (Foxp2^Fx/Fx^)  2. cHET  (Emx1-Cre; Foxp2^Fx/+^)  3. cKO  (Emx1-Cre; Foxp2^Fx/Fx^)  4. Emx1-Cre only  (Emx1-cre; Foxp2^+/+^) | Kruskal-Wallis test | 1. Foxp2Fx; N=7 mice  2. cHET; N=6  3. cKO; N=7  4. Emx-cre; N=6 | FOG2 (Fig. 3C)  p= 0.0437*  CTIP2 (Fig. 3D)  p=0.5157 |
| 3D | Pairwise comparisons of FOG2+ (CT) neurons at P0  with Dunn’s multiple correction | 1. Foxp2Fx  (Foxp2^Fx/Fx^)  2. cHET  (Emx1-Cre; Foxp2^Fx/+^)  3. cKO  (Emx1-Cre; Foxp2^Fx/Fx^)  4. Emx1-Cre only  (Emx1-cre; Foxp2^+/+^) | Dunn’s post hoc test  (6 comparisons) | 1. Foxp2Fx; N=7 mice  2. cHET; N=6  3. cKO; N=7  4. Emx-cre; N=6 | Foxp2 Fx vs. cHET p>0.9999  Foxp2 Fx vs. cKO  p=0.3688  Foxp2 Fx vs. Emx1-cre  p> 0.9999  cHET vs. cKO  p=0.0674  cHET vs. Emx1-cre  p=0.9999  cKO vs. Emx1-cre  p=0.1353 |
| 3F | Quantifcation of CCK+ cell numbers in layer 6 at P14 | 1.Foxp2Fx (Foxp2^Fx/Fx^)  2. cKO (Emx1-cre; Foxp2^Fx/Fx^) | Unpaired,  two-tailed T-test | 1. Foxp2Fx; N=3  2. cKO; N=4 | p=0.5178 |
| 3G | Quantification of FOG2+ cell numbers at P14 | 1. Foxp2Fx  (Foxp2^Fx/Fx^)  2. cKO (Emx1-cre; Foxp2^Fx/Fx^) | Unpaired, two-tailed T-test | 1. Foxp2Fx; N=3  2. cKO; N=4 | p=0.6912 |
| - | Quantification of CTIP2+ cell numbers at P14 | 1. Foxp2Fx  (Foxp2^Fx/Fx^)  2. cKO (Emx1-cre; Foxp2^Fx/Fx^ | Unpaired, two-tailed T-test | 1. Foxp2Fx; N=3  2. cKO; N=4 | p=0.99 |
| 3H | Somatosensory cortical thickness | 1. Foxp2Fx  (Foxp2^Fx/Fx^)  2. cKO (Emx1-cre; Foxp2^Fx/Fx^) | Unpaired, two-tailed T-test | 1. Foxp2Fx; N=3  2. cKO; N=4 | p=0.583 |

(For large datasets, or papers with a very large number of statistical tests, you may upload a single table file with tests, Ns, etc., with reference to sections in the manuscript.)

**Group allocation**

- Indicate how samples were allocated into experimental groups (in the case of clinical studies, please specify allocation to treatment method); if randomization was used, please also state if restricted randomization was applied
- Indicate if masking was used during group allocation, data collection and/or data analysis

Please outline where this information can be found within the submission (e.g., sections or figure legends), or explain why this information doesn’t apply to your submission:

-Experimental groups in this study were defined based on the genotype of each animal.

-All analyses (cell type quantification, axonal fluorescence analysis), whether automated or manual, were performed by observers blind to genotype.

**Additional data files (“source data”)**

- We encourage you to upload relevant additional data files, such as numerical data that are represented as a graph in a figure, or as a summary table
- Where provided, these should be in the most useful format, and they can be uploaded as “Source data” files linked to a main figure or table
- Include model definition files including the full list of parameters used
- Include code used for data analysis (e.g., R, MatLab)
- Avoid stating that data files are “available upon request”

Please indicate the figures or tables for which source data files have been provided:

-The data files have been uploaded to Dryad:

DOI: <https://doi.org/10.5061/dryad.6hd7bf7/1>

-The ImageJ macros used for semi-automated cell counting associated with Figure 3 are included in the submission as .ijm files.
